# Lessons Learnt From the COVID-19 Pandemic

**DOI:** 10.3389/fpubh.2021.694705

**Published:** 2021-08-02

**Authors:** Nils Chr. Stenseth, Guha Dharmarajan, Ruiyun Li, Zheng-Li Shi, Ruifu Yang, George F. Gao

**Affiliations:** ^1^Centre for Ecological and Evolutionary Synthesis, Department of Biosciences, University of Oslo, Oslo, Norway; ^2^Savannah River Ecology Laboratory, University of Georgia, Aiken, SC, United States; ^3^CAS Key Laboratory of Special Pathogens, Wuhan Institute of Virology, Chinese Academy of Sciences, Wuhan, China; ^4^State Key Laboratory of Pathogen and Biosecurity, Beijing Institute of Microbiology and Epidemiology, Beijing, China; ^5^CAS Key Laboratory of Pathogen Microbiology and Immunology, Institute of Microbiology, Chinese Academy of Sciences, Beijing, China; ^6^Chinese Center for Disease Control and Prevention, Beijing, China

**Keywords:** pandemic, COVID-19, epidemiology, SARS-CoV, public health

## Abstract

The COVID-19 pandemic, caused by the novel coronavirus SARS-CoV-2, has been characterized by unprecedented rates of spatio-temporal spread. Here, we summarize the main events in the pandemic's timeline and evaluate what has been learnt by the public health community. We also discuss the implications for future public health policy and, specifically, the practice of epidemic control. We critically analyze this ongoing pandemic's timeline and contrast it with the 2002–2003 SARS outbreak. We identify specific areas (e.g., pathogen identification and initial reporting) wherein the international community learnt valuable lessons from the SARS outbreak. However, we also identify the key areas where international public health policy failed leading to the exponential spread of the pandemic. We outline a clear agenda for improved pandemic control in the future.

## Introduction

In late-December 2019, pneumonia of unknown etiology (PUE) was reported from a cluster of patients who were initially linked epidemiologically to the Huanan Seafood Market in Wuhan, China (1, 2). These cases of PUE were reported to the WHO China Country Office on December 31, 2019 (3), and the Chinese Center for Disease Control and Prevention (CCDC) sent an investigative team to Wuhan on the same day. The first batch of samples was dispatched to three organizations (China CDC, Wuhan Institute of Virology under Chinese Academy of Sciences and Chinese Academy of Medical Sciences) for virus genome sequencing and virus isolation. Parallel experiments from these organizations were carried out with coordination of the National Health Commission to make sure the results were comparable. The novel SARS-related coronavirus was identified when several PUE samples tested positive with a pan-coronavirus RT-PCR covering all SARS-related coronaviruses, and the pan-PCR product was sequenced. As of January 7, 2020, Chinese health officials had confirmed that the PUE was caused by a novel coronavirus (4). Hence, it took China just about a week to inform the world about the etiology of the PUE, which is indeed efficient for identifying a novel pathogen causing an emerging infectious disease, demonstrating China's improved ability to manage new outbreaks (5). Concurrent to the virus identification, NGS-sequencing was also carried out and on 10 January, 2020, the CCDC shared the whole genome sequences through the Global Initiative on Sharing All Influenza Data (GISAID; Accession numbers EPI_ISL_402119 and EPI_ISL_402121) (6), and reported these data to the WHO. Prior to the work in early January 2020, the Wuhan Institute of Virology had sequenced similar bat-derived coronaviruses, but did not have SARS-CoV-2 in the lab suggesting a laboratory-origin as being unlikely (7), a finding supported by a recent WHO report (8). Specifically, the Wuhan Institute of Virology got the partial sequences of the *RdRp* gene by pan-coronavirus RT-PCR from a bat fecal swab collected in 2013 (sample ID 4991) and later named RaTG13 following the bat species, samples location and year. The institute ignored this sequence when they found it is distantly related to SARS-CoV. In 2018, they decided to sequence as much as possible of the full-length genome from their stock samples which are positive for SARS-related coronavirus. They completed the nearly full-length genome sequence of RaTG13 but didn't publish it. In 2020, after they received the SARS-CoV-2 sequence, the institute compared it with all of their unpublished sequences and found its closest relative RaTG13 (96.2% nucleotide identity), and they then completed the whole genome sequence (9). As of current date, the RaTG13 strain has never been isolated and has not been the focus of further studies, except with regard to ACE2 interaction using pseudovirus and binding affinity assays. The current data indicate that the TG13 spike has low binding affinity to human ACE2 compared to SARS-CoV-1, SARS-CoV-2, Pangolin CoV and bat SARS-related CoV WIV1 (10–13).

## Policy Options and Implications

### Global Response

**Figure 1:**
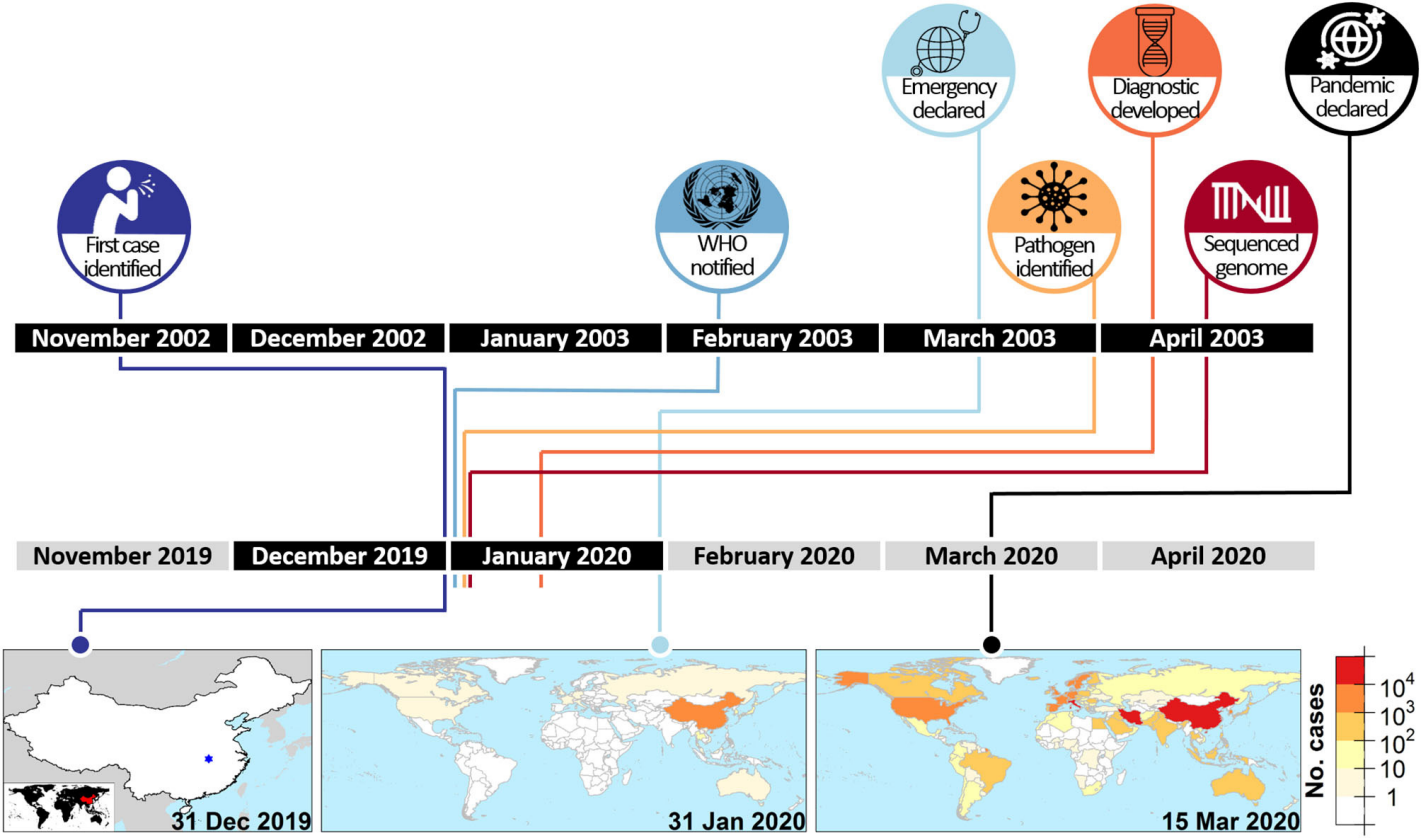
Comparative timelines of two coronavirus epidemics. The relative timelines for SARS-CoV epidemic of 2002–2003 (top row) (14–19) and SARS-CoV-2 pandemic of 2019 (bottom row) (20–22), specifically highlighting dramatic differences in duration to notification of the WHO, identification of the pathogen and sequencing of the pathogen genome after the identification of the first case. The maps show the spatial extent of SARS-CoV-2 infection at three critical time points (23, 24).

It is clear that both China and the WHO demonstrated dramatically improved responses to COVID-19 with the lessons learned from previous epidemics (see Figure 1). For example, in the case of the SARS-CoV epidemic (2002-2003), initial cases were identified in mid-November 2002 but it was only on 10 February 2003 that the developing epidemic was confirmed and the first report to WHO was made (14). In stark contrast, as highlighted above, Chinese officials informed WHO of a potential epidemic within a week of the first cases being identified in Wuhan. The WHO also acted quickly and formally alerted the world of a public health emergency of international concern (PHEIC) by end of January, well before pandemic spread had started. Indeed, at the time the PHEIC was declared only 25 countries/regions outside mainland China had reported any cases of the disease, and of these only 6 (i.e., Hong Kong, Japan, Singapore, South Korea, Taiwan, Thailand) had reported over 10 cases. Unfortunately, the rest of the world did not seem to pay too much attention to these alerts, and it was not until mid-March—when WHO Director-General announced COVID-19 as a global pandemic (25)—that the rest of the world “woke up” and started to accept that a pandemic was developing.

### Source Identification

While identifying the origin of COVID-19 is essential to prevent the next pandemic (26), the actual origin of SARS-CoV-2 remains enigmatic (8). Viruses that are phylogenetically related to SARS-CoV-2 have been identified in several wildlife species (e.g., horseshoe bats and pangolins), but as of now no wildlife species has tested positive SARS-CoV-2 across China (8). It is currently proposed that the entry of the virus into the human population could have been facilitated by cross-species transmission through one or multiple intermediate host species (9, 27, 28). However, this hypothesis is primarily based on our understanding of SARS MERS, and/or avian flu, and may need to be revisited and assessed as more data come to light. Initially it was also suspected that the coronavirus had entered the human population through the Huanan Seafood Market, a live animal market in Wuhan, China. However, the early report for COVID-19 did not find direct epidemiological links for many patients with the market (29). Thus, it is possible that the live animal markets might have served as an amplifier due to large numbers of people in close proximity to each other in the cold environment—just like the after-ski bar in Kitzloch, Austria (30).

### Control Strategies

Different strategies have been implemented to combat the pandemic in different countries. Some countries (e.g., Sweden) initially tried so-called herd immunity by natural infections and some used mitigation or suppression, but in general these approaches had minimal effects on stopping the spread of the disease within and between countries. Generally, most countries across the globe tried to limit the spread of the pathogen through various non-pharmaceutical interventions (NPIs), including the implementation of lockdowns of varying intensity and geographic scope. However, it has been noted that the inadequate (e.g., United States and India) or delayed (e.g., Russia, United Kingdom, and France) implementation of lockdowns could have reduced their efficacy in impeding the spread of infections in many countries (31). Just as delays in implementing lockdowns have increased pathogen spread, the premature lifting of these restrictions can also cause a resurgence in case numbers as has been observed recently in India (32, 33). One of the most effective implementations of NPIs was undertaken by China, which took immediate and stringent measures to prevent pathogen spread, including the lock-down of the city of Wuhan, where the virus was first identified, and suppression measures for the rest of the country (34). The WHO-China Joint Mission on COVID-19 (35) revealed that the immediate prevention and control measures that China took to curtail the epidemic were implemented in three main phases. The first stage focused primarily on preventing cases from being exported from Wuhan in conjunction with closing wet markets and enhanced surveillance to try to identify the zoonotic source. The second stage focused on controlling the impacts of the epidemic through medical intervention, improved diagnostics for rapid identification of infected individuals, and critically on reducing the rate of spread by curtailing the movement of people, restriction of mass gatherings, contact tracing, increased quarantine measures, and enhanced border security. Finally, in the third stage the focus shifted to controlling isolated and/or sporadic case clusters. In this stage there was a critical effort to strike a balance between effective disease control and sustainable economic/social development. The effective implementation of these policies made China one of the most successful countries in terms of COVID-19 control. The effectiveness of China's control measures is evidenced in terms of the per-capita cases reported. Thus, as of May 6, 2021, the global infection rate was about 20,022/million persons, with considerable variation amongst various countries (e.g., 98,503 and 15,573 cases/million persons in USA and India, respectively). However, China's cumulative infection rates remained one of the lowest globally (71 cases/million persons) (36). It is important to note that after Wuhan outbreak which was cleared on April 8, 2020 (37–39), China has experienced many small waves of outbreak with local transmission due to imported cases, but all the viruses are “stable” strains (with single imported case of both 501Y.V1 and 501Y.V2 but no local spread) and there are no new variants arising from China, indicating the successful suppression of virus circulation. For both containment and suppression strategies, lock-down of the city/region (the areas could be very small), lock-down of the household and isolation/quarantine are the three important factors for the success. Looking to the future, with no recurrent outbreaks in China even in the winter season (as of Feb 23, 2021), we might consider such a mitigation strategy to ensure meeting public health goals, while keeping the society socially active and economically strong. It is also important to recognize the need for better international coordination in terms of reducing transmission (e.g., restriction of social gatherings and mask ordinances) and the timely identification of potential spread (e.g., contact tracing). Early in the pandemic these measures, in conjunction with stricter limitations of international travel, would have helped reduce the initial global spread of the virus. However, in these late stages of the pandemic, localized lockdowns (e.g., at city or county scale) are likely to be more effective than large-scale lockdowns at national or regional levels (40).

## Actionable Recommendations

We have learnt many critical lessons from the ongoing coronavirus pandemic with respect to the requirements for rapid response and large-scale surveillance, as well as the needs for effective and coordinated strategies to control novel pathogens. While there remain many unknowns and uncertainties relating to the control of future pandemics, based on what we learnt from SARS-CoV-2 we make the following recommendations:

1. While pandemics are unpredictable by nature, proper preparation and prior planning can help manage them better. For a long time, coronaviruses have been identified as pathogens with high pandemic potential, and have thus been high on the prioritized preparedness list. Yet the globe was still unprepared to effectively deal with COVID-19. There is no doubt that many of the science-based requirements for pandemic control—rapid identification of the causative agent, genome sequence and determination of the key epidemiological parameters related to transmission—were met, but the global management of the pandemic still failed in many respects. Clearly, science alone cannot control a pandemic. In the long run, active science outreach to the public and policy makers are fundamental to achieving a coordinated implementation of intervention across scales, sectors and population groups (41). There is no doubt that a unifying science-based strategy, public involvement, and informed decision-making are the three key steps to improve the control of such public health emergencies in the future.
2. Both China and international communities outside China have learnt the vital need for improved preparedness to rapidly identify and limit the spread of emerging pathogens. Stockpiling of emergency supplies and the logistics of meeting rapidly ramped-up demand was a major bottleneck in the response to COVID-19. From the very beginning medical and public health workers were faced with the shortage of many essential items, including equipment for oxygenation support (e.g., oxygen masks, respirators, and ECMO/extracorporeal membrane oxygenation) and even personal protection equipment (e.g., face masks and gloves). Despite the lesson learnt, there seems to be no practical way to address this issue because there is no easy way to store such supplies in bulk for logistic and economic reasons. Additionally, the next pandemic may be characterized by other symptomatology (e.g., hemorrhagic fever) rather than respiratory failure. Thus, there is a need to think of creative solutions to address our ability to meet such sudden supply-demand dynamics in the future, and we would like to leave this as an open question to the readers.
3. The WHO has to be given a much stronger role in the coordination of the implementation of the various control-measures. Given the exponential nature of pathogen spread it is imperative that we ensure the rapid mobilization of mitigation and control strategies at international scales before local epidemics can progress to pandemics. The authority of the WHO for global coordination of pandemic responses must be strengthened.
4. There is also an urgent need to address several open questions related to COVID-19, particularly the possible reservoir or intermediate host(s), the role of live-animal markets in introducing or maintaining the virus in the human population. China-WHO Joint Study Group recently spent a month (January-February, 2021) in Wuhan to investigate the origins of the virus, but with no definitive answers yet. For the whole year of 2020, scientists and public health professionals in China and across the globe have been trying to answer this critical question, but with limited success. For example, it still remains unclear if SARS-CoV-2 differs fundamentally from other coronaviruses (e.g., SARS-CoV and MERS-CoV) in terms of its epidemiology and entry into the human population (42). Given the lack of information there is a necessity to keep an open mind and follow an objective scientific agenda to address the outstanding questions.
5. It is encouraging that shortly after the genome was made publicly available identification of potential vaccine candidates was initiated, with NIH joining up with Moderna Inc. in mid-January (43). Potential vaccine candidates were rapidly screened, with Moderna publishing their preliminary report on the mRNA-based COVID-19 vaccine on 14 July 2020 (44), and BioNTech and Pfizer publishing safety and immunogenicity data from Phase 1 clinical trials of two RNA vaccine candidates on October 14, 2020 (45). Given that several vaccines have now been approved for human use, it is clear that vaccine development for COVID-19 has progressed at an extremely rapid rate, with less than a year elapsing from initial pathogen discovery to vaccine deployment. There is no doubt that the rapid development of the SARS-CoV-2 vaccine was only possible because it was able to leverage a large body of basic research on other coronaviruses, such as MERS-CoV (46). Such an approach to preparedness using prototype pathogens could also be started for the other 23 virus families known to infect humans (e.g., Flaviviridae and Filoviridae), thus dramatically improving our ability to manage future pandemics (43).
6. While vaccines are a key component of control, the recent pandemic has also highlighted the fundamental importance of NPIs given their efficacy in reducing viral spread (47). While the effectiveness of these NPIs is highly variable (e.g., depending on community-level infection rates), several specific NPIs have been shown to consistently reduce the transmission of SARS-CoV-2. These interventions include the closure of schools and workplaces, bans on public events and gatherings of more than 10 people, as well as limiting human movement (48). The continued use of such NPIs needs to be enforced especially at the very early stages of vaccination.
7. Most importantly, an international scientific conference should be convened, as soon as possible, to discuss not only the biomedical issues related to the pandemic, but also other issues related to pandemic control, such as the effectiveness of the various interventions adopted in different countries (47) and the need for improved policy coordination (49). Such large international conferences have been very effective in the past. A historic example includes the international sanitary conference held in February 1897 at Venice to discuss the spread of plague (50), and the international conference following the Manchurian plague of 1910-1911 in Shenyang (then Fengtian), China (50–53). These examples can serve as portfolios that can be effectively adopted to better evaluate the strengths and weaknesses of country-specific and international responses to the COVID-19 pandemic and to improve pandemic response in the future.

## Conclusions

The ongoing COVID-19 pandemic caused by SARS-CoV-2 has brought to the fore the devastating societal and economic consequences associated with emerging infectious diseases. Human history has been punctuated by many such global pandemics including the bubonic plague (14th century), the flu (20th century) and HIV/AIDS (20th and 21st century), and it is unlikely that COVID-19 will be the last one. Indeed, the risk of emergence of novel diseases in human populations is increasing at an alarming rate due to numerous factors including the rapid range expansion of disease vectors, destabilization of natural ecosystems, as well as the rapid increase in agriculture and urbanization. By critically comparing the 2002–2003 SARS outbreak and COVID-19 pandemic, we identified that significant strides have been made in terms of rapid pathogen identification and expedited initial outbreak reporting by China, as well as the PHEIC declaration by WHO. However, one of the major failures was the delayed international response to the PHEIC declaration by the WHO, a delay which allowed for the exponential spread of the pandemic. We recommend that these are the critical areas that the international public health community must focus on to better control future pandemics in a highly connected global population. In the end we feel that the most essential lesson we can learn from COVID-19 is that pandemic control hinges on rapid, effective, coordinated and sustained pandemic response at local, national and international levels.

## Author Contributions

NS developed the idea. GD and NS wrote the first draft of the manuscript. GD coordinated input from other authors. All other authors contributed to specific sections, discussed results, and edited the manuscript.

## Conflict of Interest

The authors declare that the research was conducted in the absence of any commercial or financial relationships that could be construed as a potential conflict of interest.

## Publisher's Note

All claims expressed in this article are solely those of the authors and do not necessarily represent those of their affiliated organizations, or those of the publisher, the editors and the reviewers. Any product that may be evaluated in this article, or claim that may be made by its manufacturer, is not guaranteed or endorsed by the publisher.
